# Rituximab Therapy in Pulmonary Alveolar Proteinosis: A Case Report

**DOI:** 10.7759/cureus.73836

**Published:** 2024-11-17

**Authors:** Oussama Fikri, Meryem Hindi, Lamyae Amro

**Affiliations:** 1 Department of Pulmonology, Centre Hospitalier Universitaire (CHU) Mohammed VI, Arrazi Hospital, Faculté de Médecine et de Pharmacie de Marrakech, Laboratoire de Recherche Morpho Sciences, Université Cadi Ayyad (FMPM, Labo LRMS, UCA), Marrakech, MAR

**Keywords:** crazy paving, pulmonary alveolar proteinosis, pulmonary biopsy, rituximab, surfactant

## Abstract

Autoimmune pulmonary alveolar proteinosis (PAP) is a rare lung condition characterized by the accumulation of surfactant proteins within the alveoli, leading to respiratory distress. We describe a 49-year-old female homemaker with a history of passive smoking and exposure to wood smoke and pigeon droppings. She presented with a dry cough and progressive dyspnea, experiencing significant deterioration in her condition over one year. Chest imaging revealed bilateral alveolar-interstitial syndrome with ground-glass opacities and alveolar condensations. Biopsy findings indicated type II pneumocytes and eosinophilic material, confirming a diagnosis of autoimmune PAP. After ruling out secondary causes of PAP, rituximab was administered successfully, leading to marked improvement in respiratory function and significant regression of radiological lesions within two months. This case highlights the importance of early diagnosis and treatment of autoimmune PAP, demonstrating the potential efficacy of rituximab in managing this challenging condition.

## Introduction

In 1958, Rosen et al. [1] initially described that pulmonary alveolar proteinosis (PAP) is a rare pulmonary disease that affects people all over the world, characterized by an accumulation of surfactant-derived proteins and lipids in the pulmonary alveoli, often as a result of defective surfactant elimination by alveolar macrophages [2], which affects gas exchange leading to respiratory failure.

Three groups of PAP are distinguished: autoimmune PAP, secondary PAP, and genetic PAP. The clinical features of PAP include an insidious onset with an indolent course of symptoms, such as asymptomatic presentation, dyspnea on exertion, cough, sputum production, fatigue, weight loss, and low-grade fever. Physical examination may initially be normal, but some patients can have crackles and cyanosis [3].

Whole-lung lavage is the standard of care and may lead to a number of complications [4]. Other treatments are based on the supplementation of granulocyte-macrophage colony-stimulating factor (GM-CSF), which only proves efficient in nearly 50% of cases [5]. Rituximab is a monoclonal antibody directed against the CD20 antigen of B-lymphocytes. In autoimmune diseases, rituximab reduces the production of antibodies, such as anti-GM-CSF antibodies by declining B-lymphocytes.

The presented clinical case highlights the complexities and challenges associated with diagnosing and managing autoimmune PAP. This patient's unique presentation, with significant respiratory compromise and rapid clinical deterioration, underscores the importance of early recognition and intervention. The initiation of rituximab therapy illustrates a novel and effective approach to treatment, especially in cases refractory to standard therapies. Furthermore, this case contributes to the growing body of evidence supporting the use of targeted therapies in autoimmune PAP, emphasizing the need for personalized treatment strategies that address the underlying pathogenic mechanisms of the disease.

## Case presentation

The patient is a 49-year-old homemaker, who was never treated for tuberculosis and exposed to passive smoking, wood smoke and pigeon droppings for two years; she had been vaccinated against COVID-19 and had no previous COVID-19 infection.

She had been suffering for one year from a dry cough, SADOUL stage II dyspnea that had become stage V for 30 days, and sleep deprivation. Field auscultation revealed bilateral basithoracic crackles and digital hippocratism of the fingers. The patient was initially treated with azithromycin, short-term corticosteroid therapy, and vitamin supplementation due to a suspected COVID-19 infection.

**Figure 1:**
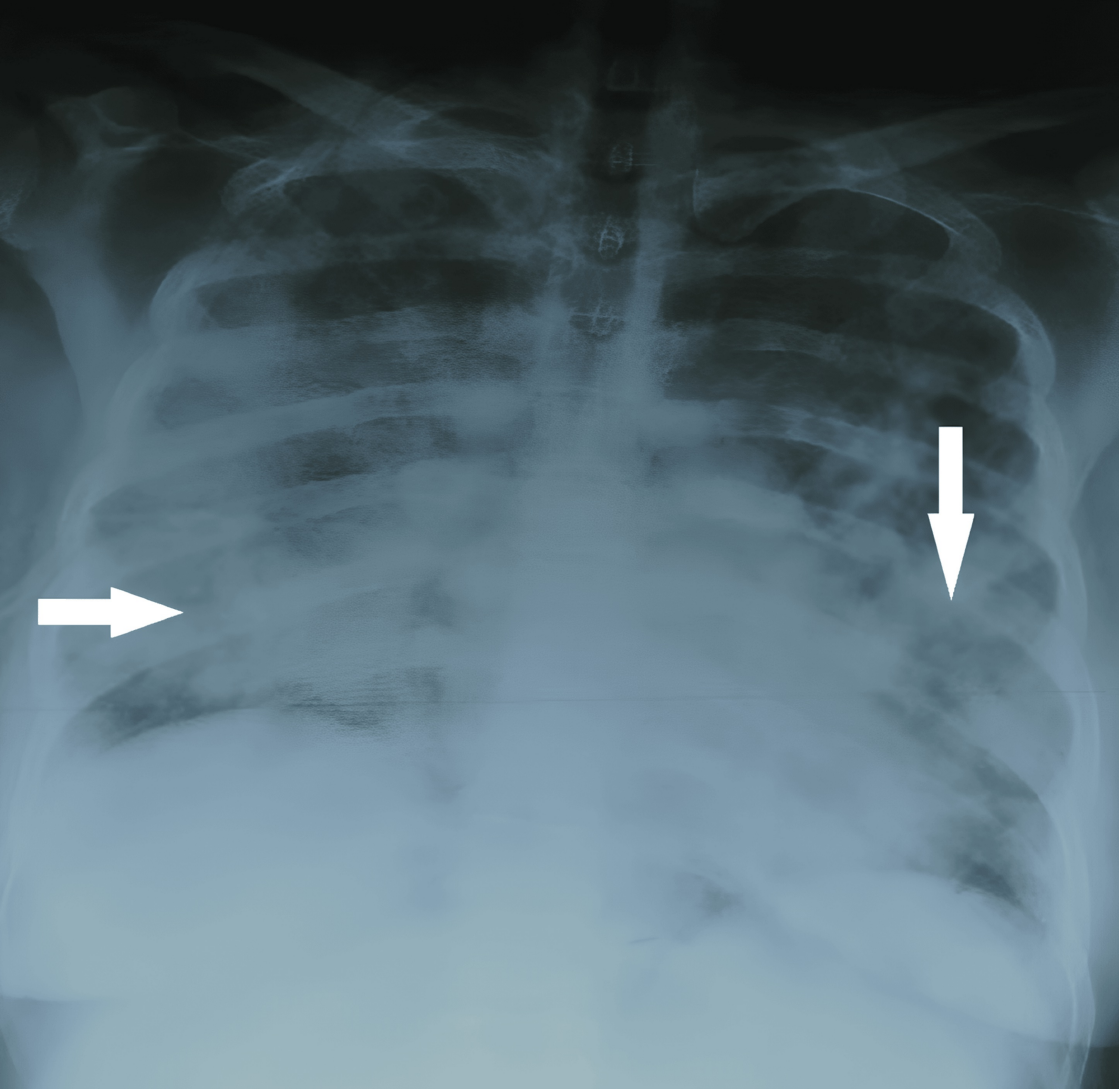
Chest X-ray showing bilateral alveolo-interstitial syndrome with predominantly peri-hilar micronodular reticulations.

**Figure 2:**
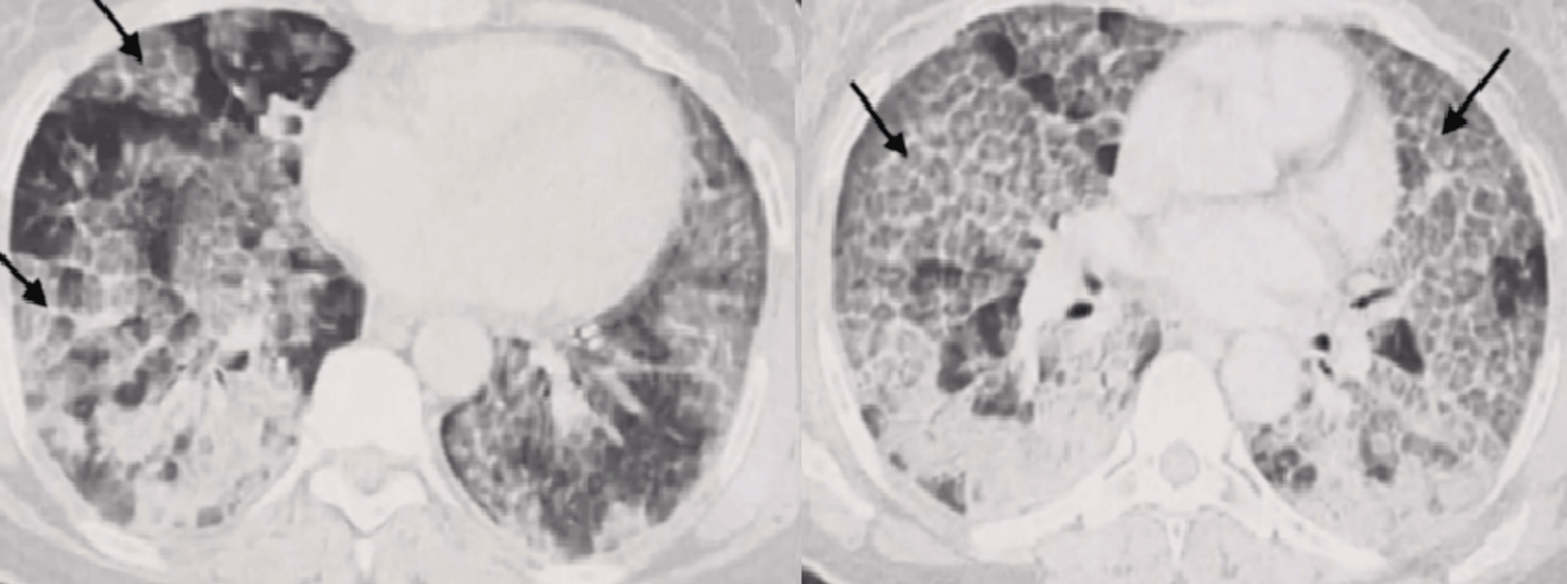
Thoracic CT scan shows the characteristic "crazy paving" appearance in a geographic distribution associated with alveolar parenchymal condensations, with thickening of the inter- and intralobular septal lines.

Chest X-ray showed a bilateral alveolar-interstitial syndrome with predominantly peri-hilar micronodular reticulations (Figure 1). In the absence of clinical improvement, a thoracic CT scan was ordered, revealing diffuse ground-glass opacities, giving the appearance of crazy paving, associated with alveolar parenchymal condensations (Figure 2).

The different considered diagnoses were infection in particular pneumocystis, hypersensitivity pneumonia, PAP, eosinophil pneumonia, exogenous lipid pneumonia and tumors including invasive mucinous carcinoma.

Bird breeder serum precipitin, immunological tests, COVID-19 PCR and HIV serology were negative, protein electrophoresis shows hypogammaglobulinemia, lactate dehydrogenase (LDH) in serum was elevated to 727, and the serum anti GM-CSF immunoglobulin (Ig)G concentration not available. The patient had respiratory instability, so there was no bronchoscopy done.

**Figure 3:**
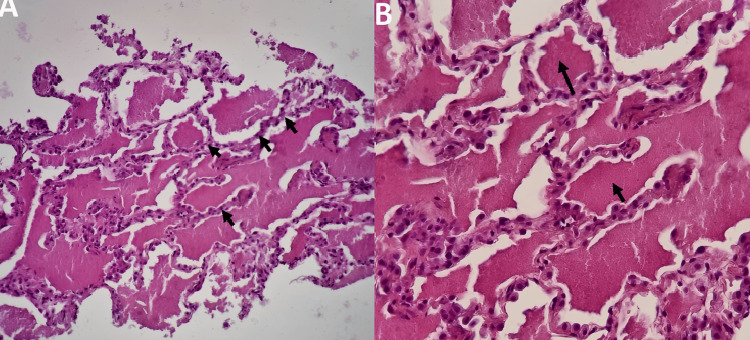
Histological appearance of PAP on CT scan-guided lung biopsy. A: Alveolar spaces lined with predominantly type II pneumocytes. B: Under high power, a proteinaceous material in the alveoli is seen.

A CT-guided biopsy was performed without incident of pneumothorax or alveolar hemorrhage yielding four biopsy cores, revealing lung parenchyma with alveolar spaces lined by predominantly type II pneumocytes, and interstitial tissue with a discrete inflammatory infiltrate of mononuclear elements. The alveolar lumen is filled with an amorphous eosinophilic material and a few foamy histiocytes (Figure 3).

The arterial blood gas analysis revealed severe type I respiratory failure, with a PaO2 of 37 mmHg and an SaO2 of 71%. Due to respiratory instability, plethysmography and diffusing capacity of the lungs for carbon monoxide (DLCO) were not performed. After ruling out secondary causes of PAP, a diagnosis of autoimmune PAP was confirmed. Over a two-month period, the patient's clinical condition progressively deteriorated. In addition to the radiological decline, the patient experienced increased dyspnea and desaturation, with oxygen levels falling to 54% despite well-adapted ventilatory support. Given the impossibility of performing a whole lung lavage due to hemodynamic and respiratory instability, the patient consented to a trial of rituximab treatment after receiving an information sheet outlining the potential benefits and risks and subsequently signing an informed consent form.

**Figure 4:**
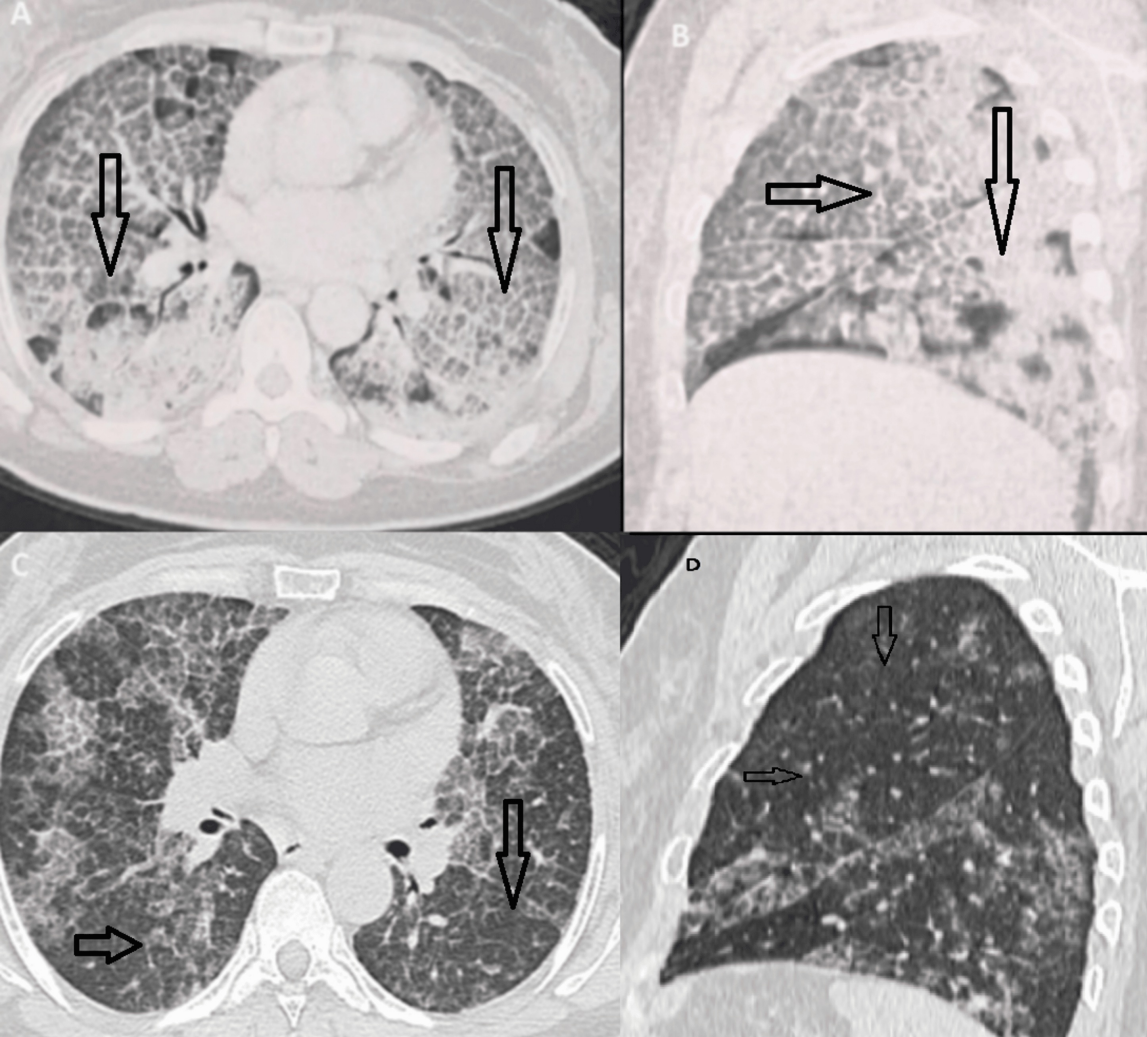
High-resolution computed tomography (A, B) before and (C, D) two months after rituximab infusion. Computed tomography images two months after rituximab treatment (C and D) showed a significant reduction in diffuse ground-glass opacification in the alveoli.

Before starting treatment, a comprehensive pre-treatment evaluation was conducted to rule out any contraindications. This included tests such as complete blood count (CBC), blood ionogram, urea, creatinine, ASAT, ALAT, GGT, alkaline phosphatases, bilirubin, CPK, LDH, CRP, plasma protein electrophoresis, serum IgG, IgA, and IgM assays, as well as HIV and hepatitis B and C serologies, and an electrocardiogram (ECG). Premedication with an antipyretic and antihistamine was also given to minimize potential side effects. Rituximab was administered in April 2023, with a dosage of 1,000 mg delivered intravenously on both day 1 and day 15. The treatment was well tolerated, leading to significant improvement in respiratory function, as shown in Table 1, and notable regression of radiological lesions two months post-treatment, as depicted in Figure 4.

**Table 1:** Lung function testing before and after rituximab infusion DLCO: Carbon monoxide diffusing capacity, TVR: Restrictive ventilatory disorder, PaCO2: Carbon dioxide blood pressure, PaO2: Oxygen blood pressure, SaO2: Arterial oxygen saturation, 6MWT: 6-minute walk test.

		Avant RITUXIMAB	1 mois	3 mois
DLCO %		-	45 %	47 %
TVR %		-	47 %	67 %
PaCO2		40.9	34.5	39
PaO2		37	57.6	65.9
SaO2		71 %	90.8 %	92.7 %
6MWT	Resting saturation-	64 %	94 %	94%
	Lowest saturation on 6MWT	49 %	85 %	86%
	Walk distance m	30 m	450 m	480 m

## Discussion

Rituximab has been shown to deplete human B-cells in vivo. It is a chimeric murine-human monoclonal antibody directed against CD20, a B-lymphocyte-specific membrane antigen [6]. After receiving rituximab, B-lymphocyte depletion in peripheral blood happens quickly within days. Rituximab is thought to kill off B cells through a variety of mechanisms, including complement-mediated cytotoxicity, antibody-dependent cell cytotoxicity, and antibody-mediated apoptosis [7].

The US Food and Drug Administration (FDA) first used rituximab to treat CD20+ B-cell lymphoma in 1997; it is now also used to treat other B-cell malignancies. Rituximab has recently been used to treat autoimmune disorders and has demonstrated clinical efficacy in a number of conditions, including systemic lupus erythematosus, rheumatoid arthritis, and granulomatosis with polyangiitis [8].

The first study to describe the effectiveness of rituximab in a patient with autoimmune PAP who refused GLPT was conducted by Borie et al. [9]. The use of rituximab therapy improves alveolar macrophage function in patients with autoimmune PAP by reducing anti-GM-CSF antibody levels and promoting resorption of lipoprotein material in the lungs [10]. This improvement can be observed even in the presence of a partial decrease in circulating anti-GM-CSF neutralizing activity and anti-GM-CSF IgG concentration in serum [10].

Kavuru et al. subsequently published a case series of 10 patients treated with rituximab, with improvement in PaO2, EFR and chest imaging in 70% of cases [8]. The study also showed a reduction in anti-GM-CSF immunoglobulin G (IgG) levels in the study participants' bronchoalveolar lavage fluid. Accordingly, lowering anti-GM-CSF autoantibodies may be crucial for enhancing PAP symptoms. High-resolution CT scan improvements were correlated with autoantibody levels in bronchoalveolar lavage fluid, indicating a link between autoantibody levels in the target organ and disease pathophysiology [8].

The proposed treatment regimen is two intravenous injections of rituximab (1 gram) at 15-day intervals [8,9]. After month 12, rituximab infusion can be repeated if lung opacities recur despite objective improvement in treatment. It is not always necessary to start a new rituximab infusion if there is only a slight increase in the number of circulating B lymphocytes or the anti-GM-CSF antibody titer.

This observation supports the idea that rituximab could be used as an alternative treatment for people with autoimmune PAP. It affects the maintenance of lipid homeostasis in alveolar macrophages in PAP patients. Additionally, rituximab therapy was linked to clinical lung improvement in terms of gas exchange (PaO2 on room air) and radiographic disease signs. In summary, our study concluded that rituximab was well tolerated and effective in the treatment of autoimmune PAP.

## Conclusions

Although PAP is a rare disease, progress in recent years has led to a better understanding of its mechanisms, especially in its autoimmune form, including rituximab. Nonetheless, the exact pathophysiology of the disease remains unclear. There is a need for further research into PAP. This case emphasizes the significance of including PAP in the differential diagnosis when patients present with persistent, unexplained respiratory symptoms and disease-related imaging findings. Patients with PAP may experience better outcomes and a higher quality of life with early diagnosis and appropriate treatment.
